# Genome-wide association mapping of quantitative trait loci for chalkiness-related traits in rice (*Oryza sativa* L.)

**DOI:** 10.3389/fgene.2024.1423648

**Published:** 2024-07-10

**Authors:** Qing Xu, Jianhua Jiang, Chunyu Jing, Changmin Hu, Mengyuan Zhang, Xinru Li, Jiaming Shen, Mei Hai, Ying Zhang, Dezheng Wang, Xiaojing Dang

**Affiliations:** ^1^ Institute of Rice Research, Anhui Academy of Agricultural Sciences, Hefei, China; ^2^ College of Agronomy, Anhui Agricultural University, Hefei, China

**Keywords:** candidate gene, chalkiness, genome-wide association study, molecular marker, quantitative trait loci, single-nucleotide polymorphism

## Abstract

Grain chalkiness directly affects the commercial value of rice. Genes related to chalkiness reported thus far have been discovered in mutants, but it has not been identified whether these genes can be used to improve rice quality by breeding. Therefore, discovering more quantitative trait loci (QTLs) or genes related to chalkiness in the rice germplasm is necessary. This study entails a genome-wide association study on the degree of endosperm chalkiness (DEC) and percentage of grains with chalkiness (PGWC) by combining 1.2 million single-nucleotide polymorphisms (SNPs) with the phenotypic data of 173 rice accessions. Thirteen QTLs for DEC and nine for PGWC were identified, of which four were detected simultaneously for both DEC and PGWC; further, *qDEC11/qPGWC11* was identified as the major QTL. By combining linkage disequilibrium analysis and SNP information, *LOC_Os11g10170* was identified as the candidate gene for DEC. There were significant differences among the haplotypes of *LOC_Os11g10170*, and the Hap 1 of *LOC_Os11g10170* was observed to reduce the DEC by 6.19%. The qRT-PCR results showed that the gene expression levels in accessions with high DEC values were significantly higher than those in accessions with low DEC values during days 21–42 after flowering, with a maximum at 28 days. These results provide molecular markers and germplasm resources for genetic improvement of the chalkiness-related traits in rice.

## Introduction

Chalkiness of rice refers to a white opaque part formed by loose deposition of starch and protein grains in the endosperm during the grain filling stage; it is negatively correlated with the appearance quality of rice and holds a certain relevance to the milling, eating, and cooking quality of rice (Chen et al., 2018). Chalkiness has always played an important role in the development of the rice industry. The degree of endosperm chalkiness (DEC) and percentage of grains with chalkiness (PGWC) are measures that directly affect the commercial value of rice; therefore, these are not only important indexes for evaluating rice chalkiness but also the main indexes for measuring the rice quality evaluation grade standard. High-chalkiness rice has obviously inferior mechanical strength than normal rice and easily breaks during processing, seriously affecting the quality, increasing the production cost, and reducing the market competitiveness of rice (Yoshioka et al., 2007). The taste of high-chalkiness rice after cooking can be significantly different from that of normal rice, in addition to its decreased viscosity and elasticity. Therefore, reducing rice chalkiness to improve rice quality is one of the important problems when breeding rice for high yield and good quality.

Chalkiness is a quantitative trait that is controlled by multiple genes with an obvious additive effect, and its inheritance is affected by the cytoplasmic genotype (Zheng et al., 2012). It also has maternal and endosperm effects (Shi et al., 2002). To the best of our knowledge, at least 46 quantitative trait loci (QTLs) controlling the DEC have been reported in literature (Tan et al., 2000; Zeng et al., 2002; Li et al., 2004; Wan et al., 2005; Liu et al., 2007; Chen et al., 2022), which were distributed on all 12 chromosomes of rice (Supplementary Table S1). Additionally, at least 84 QTLs controlling the PGWC have been reported (Li et al., 2003; Wan et al., 2005; Liu et al., 2007; Zhou et al., 2009; Wang et al., 2011; Zheng et al., 2012; Peng et al., 2014; Mirsa et al., 2021) (Supplementary Table S1), which were also distributed on all 12 chromosomes; of these 84 QTLs, 16 were found to be located on chromosome 6. Among these QTLs, only the *Chalk5* QTL has been finely mapped further; its function has also been confirmed to encode a vacuolar H^+^-translocating pyrophosphatase that influences the grain chalkiness of rice (Li et al., 2014).

In recent years, several related studies on the flo-series chalkiness mutants have revealed numerous possible chalkiness production pathways and mechanisms. These genes are divided into three main categories. The first group is related to energy production and distribution of mutant genes; the *Flo4* (Kang et al., 2005), *Ogr1* (Kim et al., 2009), *LOC_Os03g19890* (Jung et al., 2015), *Flo10* (Wu et al., 2019), *Flo12* (Zhong et al., 2019), *Flo13* (Hu et al., 2018), *Flo14* (Xue et al., 2019), *Flo16* (Teng et al., 2019), *Flo18* (Yu et al., 2021), and *Flo19* (Lei et al., 2022) genes can directly or indirectly affect normal energy production in the grains. The *Gif1* (Wang et al., 2008) and *Nf-YB1* (Bai et al., 2016; Xu et al., 2016) genes can affect the source–sink relationship of nutrient transport directly or indirectly. The second category entails mutant genes related to starch synthesis; the *Sbe* (Yano et al., 1985), *Wx* (Mikami et al., 1999), *SbeIIb* (Tanaka et al., 2004), *Flo5* (Ryoo et al., 2007), *Pho1* (Satoh et al., 2008), *Rsr1* (Fu and Xue, 2010), *Agpl2* (Zhang et al., 2012), *bZIP58* (Wang et al., 2013), *Flo6* (Peng et al., 2014), *Flo8* (Long et al., 2017), *Flo20* (Yan et al., 2022), *Flo22* (Yang et al., 2023), and *Flo23* (Chen et al., 2023) genes can affect the normal starch synthesis in grains directly or indirectly. The third category includes mutant genes related to starch structure formation; the *Rab5a* (Wang et al., 2010), *Pdil1-1* (Han et al., 2012), *Gpa3* (Ren et al., 2014), *Vps9a* (Wen et al., 2015), and *Bip* (Yang et al., 2022) genes have been shown to induce a chalky phenotype by affecting the proteosome and vesicle systems, resulting in abnormal filling and accumulation of intracellular starch. There are a few other mutant genes related to chalkiness, such as *Flo2* (Hikaru and Takeshi, 1981; She et al., 2010), *Flo7* (Zhang et al., 2016), *Flo11* (Zhu et al., 2018; Tabassum et al., 2020), and *Flo15* (You et al., 2019), whose formation mechanisms have not been revealed fully. The genes related to chalkiness reported so far have been discovered from mutants, but it is not clear whether these genes can be used to improve rice quality by breeding. Therefore, discovering more QTLs or genes related to chalkiness in the rice germplasm is expected to provide not only the molecular basis but also the material basis for improving rice quality.

In this study, the phenotype values of the DEC and PGWC traits were investigated in 173 rice materials across two environments (E1 and E2). By combining the resequence data, a genome-wide association study (GWAS) was performed to identify the QTLs associated with DEC/PGWC and to predict the candidate genes. These results are expected to provide new gene resources and insights into the molecular mechanisms of DEC and PGWC as well as the means to genetically improve rice quality.

## Materials and methods

### Plant materials and field planting

A total of 173 rice accessions reported previously by Hu et al. (2022) were selected and subjected to phenotype measurements. These accession names, origins, subpopulations, and sequence read archive (SRA) accession numbers from the NCBI database are listed in Supplementary Table S2. These experimental varieties were planted at the Experimental Station of Anhui Academy of Agricultural Sciences (Hefei, Anhui Province, China) in 2021 and 2022. The field management followed routine operations. Each variety was planted twice repeatedly following a completely random design; there were 36 plants for each variety at each repetition.

### Trait investigation

The grains were harvested 45 days after heading and air-dried under natural conditions for 3 months before carrying out the related experiments. Each experiment was performed twice, and the mean value of each variety from the two replicates was used to calculate its DEC and PGWC. For each repetition, about 50 whole milled rice samples were selected from each variety and placed on the glass plate of a MRS-9600 TFU2L Microtek scanner (Shanghai Zhongjing Technology Co., Ltd., Shanghai, China). These samples were then scanned using the Microtek ScanWizard EZ software. Using a rice appearance quality detection software, the proportion of the opaque parts was calculated to obtain the DEC and PGWC through the following formulas:

$$\text{PGWC }\left(\%\right)=\left(\text{Number of chalky rice grains}\right)\;/\;\left(\text{The total number of rice grains}\right)\times 100\%.$$

$$\text{DEC }\left(\%\right)=\left(\text{PGWC}\right)\times \left(\text{Chalky size}\right).$$

### Genotypic data obtained

The sequence data of the 173 accessions were downloaded from the NCBI SRA with the accession number PRJNA554986 (Dang et al., 2020). The software Bowtie 2 (Li and Durbin, 2010) was then used to align all the paired-end sequences, for which the sequence of Os-Nipponbare-IRGSP 1.0 was used as the reference sequence. More than 95% of the reads were mapped to the reference genome with a mapping score exceeding 60. The HaplotypeCaller from GATK 3.8-0 was used to retrieve the single-nucleotide polymorphisms (SNPs). The software Beagle v4.1 (Browning and Browning, 2009) was used to complete the missing genotype data, and a total of 1,224,254 SNPs with minor allele frequencies of more than 5% and missing rates below 20% were selected to perform the GWAS.

### Genome-wide association study

The compressed mixed linear model (MLM) was employed in the GWAS using the software GAPIT v.2.12 (Lipka et al., 2012). The “qqman” package of R software was used to draw the Manhattan plot (Turner, 2014). The significance threshold was set at 1.0 × 10^-5^, and the significant associated SNPs were determined on the basis of the correction method proposed by Benjamini and Hochberg (1995). The linkage disequilibrium (LD) analysis was carried out with the software Haploview 4.2, and the LD blocks surrounding the significant SNPs were detected (Barrett et al., 2005). The LD heatmap was then constructed using the “LDheatmap” program of R package (Shin et al., 2006). According to the method reported by Huang et al. (2021), there were more than three significant SNPs that exceeded the *p*-value threshold with clear peak-like signals within a 200-kb region of the leading SNP (having the smallest *p*-value), and this region was considered one QTL.

### Candidate gene analysis

Using the Rice Genome Annotation Project MSU7 database (http://rice.plantbiology.Msu.edu), the candidate genes within the 200-kb genomic region were predicted. The causal genes were then determined based on gene annotation and expression analysis.

### Quantitative real-time polymerase chain reaction (qRT-PCR) analysis of the candidate genes

After heading, the flowered spikelets of each day were marked. The spikelets marked for 7, 14, 21, 28, 35, and 42 days after flowering were sampled from three samples each having the highest and lowest DEC values. The TianGen Pure Plant Plus Kit (TianGen Biotech Co., Ltd., Beijing, China) was used to extract the total RNA. According to the instructions, the HisScript II Reverse Transcriptase system (Vazyme Biotech Co., Ltd., Nanjing, China) was used to synthesize the first-strand cDNA; the UBQ rRNA gene was used as the internal control. The qRT-PCR was then performed using SYBR Green (Vazyme Biotech Co., Ltd., Nanjing, China) based on the 96-well thermocycler (Roche Applied Science LightCycler 480, https://lifescience.roche.com/). The PCR procedures were performed in accordance with the description reported by Hu et al. (2022). For each sample, three biological repetitions were carried out, and the primers for the qRT-PCR are listed in Supplementary Table S3. The transcript levels of the gene expressions were calculated by 2^−ΔΔCt^, where ΔCt = Ct_target gene_ - Ct_UBQ rRNA_ (Livak and Schmittgen, 2001).

### Haplotype analysis

The haplotypes of the candidate genes were determined in accordance with the RiceVarMap (http://ricevarmap.ncpgr.cn/) and China Rice Data Center (https://www.ricedata.cn/) databases. Each haplotype was confirmed by at least 20 accessions.

## Results

### Phenotypic analysis of chalkiness-related traits

**FIGURE 1:**
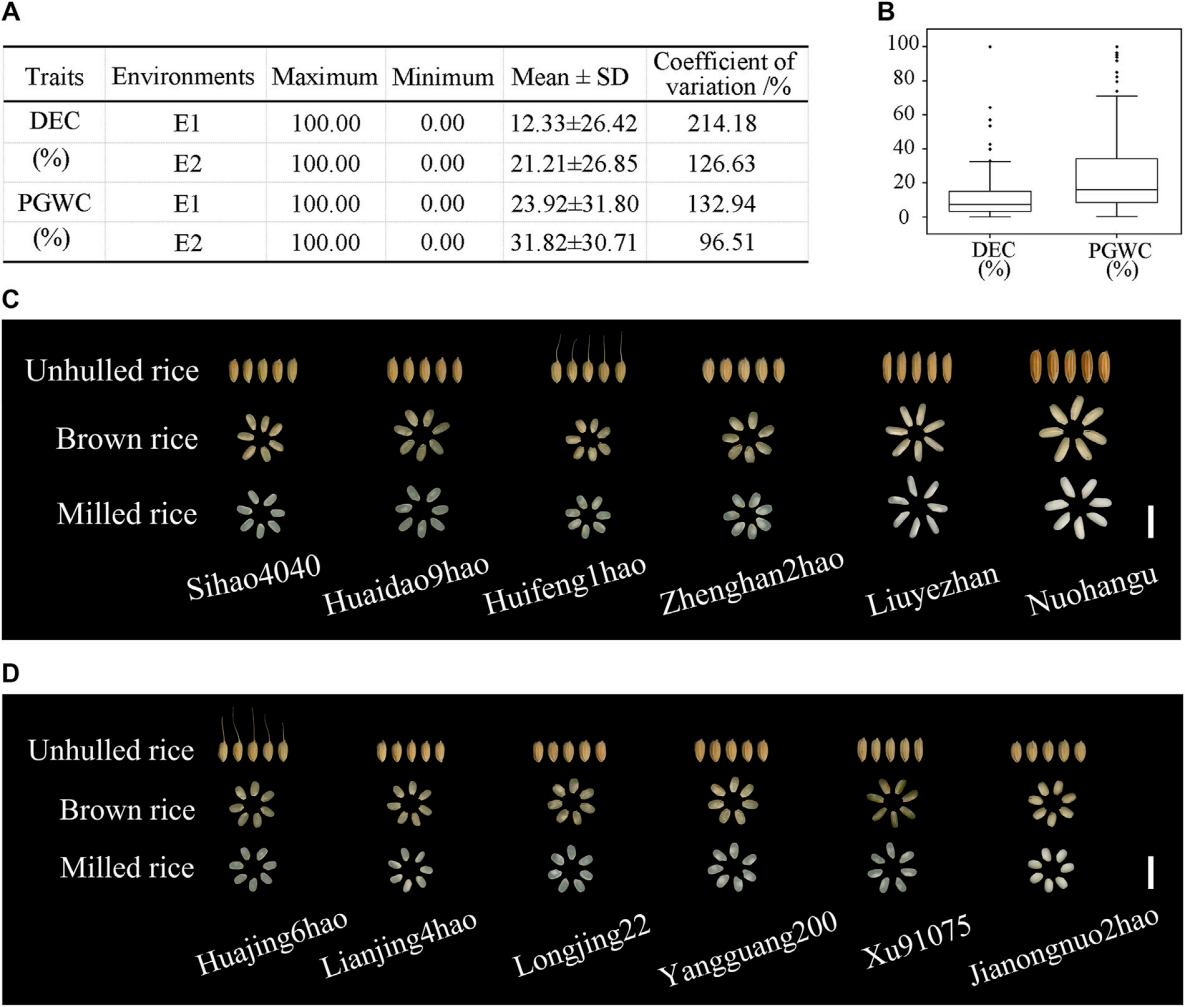
Phenotypic value descriptions of the degree of endosperm chalkiness (DEC) and percentage of grains with chalkiness (PGWC) traits among 173 rice accessions from two environments. **(A)** Basic statistics of the DEC and PGWC traits in the two environments. **(B)** Phenotypic value distributions of the DEC and PGWC traits in the two environments. The box edges indicate the upper and lower quantiles, and the lines in the middle of the boxes indicate the median values. The vertical lines indicate the data from the lowest quantile to the top quantile. **(C)** DEC and **(D)** PGWC performances in cultivars. Scale bar, 1 cm.

For the DEC trait, the mean values were 12.33% ± 26.42% in E1 and 21.21% ± 26.85% in E2 (Figure 1A), with the mean coefficient of variation (CV) being 170.41% across the two environments (Figure 1A). For the PGWC trait, the maximum value was 100% while the minimum value was 0 between the two environments (Figure 1A). Figure 1B shows the distributions of the DEC and PGWC among the 173 accessions. Figure 1C displays the milled rice morphologies of the materials with different DEC values, where the accession Nuohangu shows the maximum DEC (100%) and accession Sihao4040 shows the minimum DEC (0.15%). Figure 1D shows the milled rice morphologies of the materials with different PGWC values, where the accession Jianongnuo2hao shows the maximum PGWC (100%) and accession Huajing6hao shows the minimum PGWC (0.59%). There were abundant variations in the chalkiness-related traits of the 173 rice accessions, which provided the material basis for mining the elite allele variations.

### Identification of QTLs for chalkiness-related traits by GWAS

**FIGURE 2:**
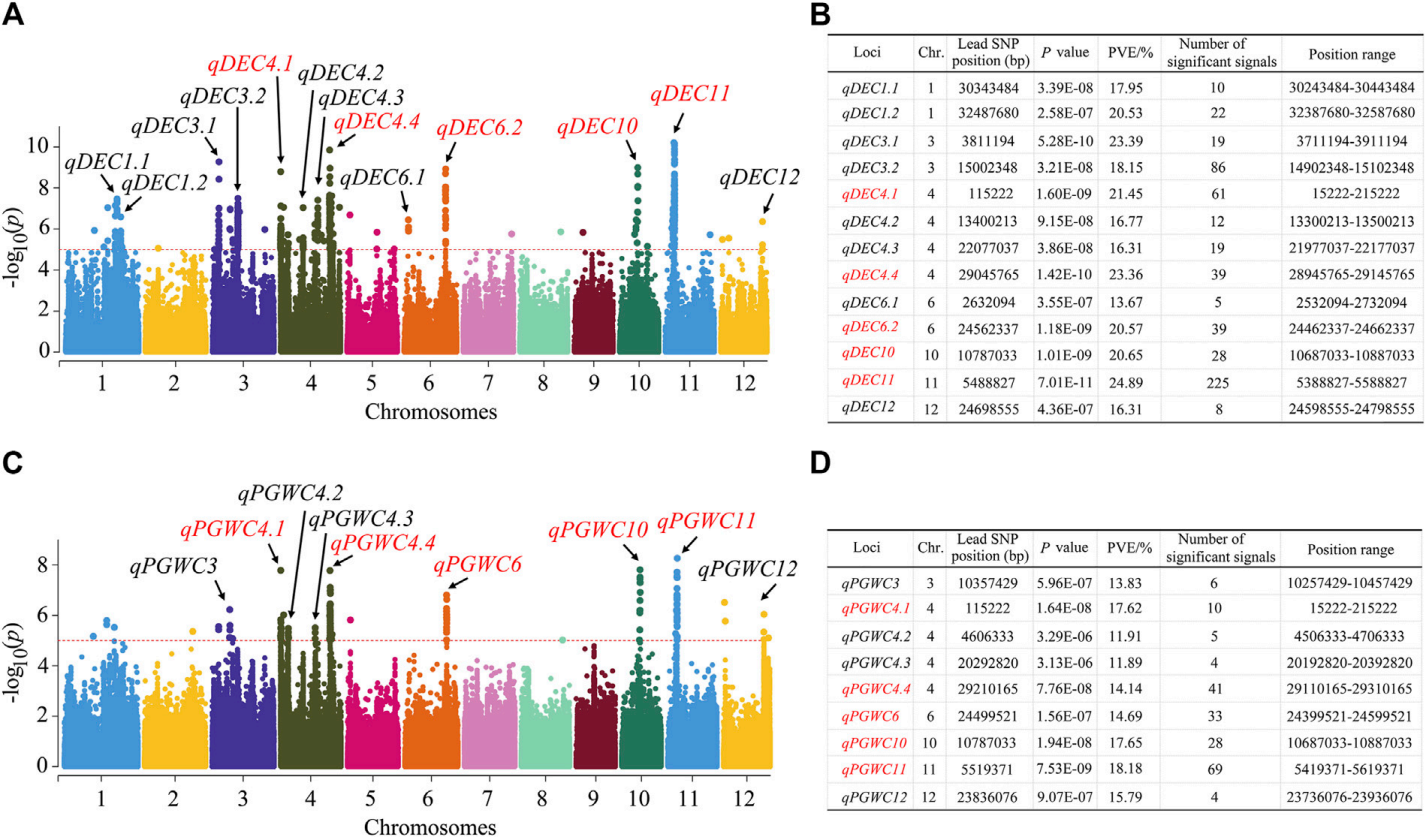
Quantitative trait loci (QTLs) identified in rice for the DEC and PGWC by the genome-wide association study (GWAS). **(A)** Manhattan plots for the DEC of the entire population of 173 rice accessions. **(B)** Information on the identified QTLs for DEC. **(C)** Manhattan plots for the PGWC of the entire population of 173 rice accessions. **(D)** Information on the identified QTLs for PGWC. Negative-log_10_-transformed *p*-values are plotted on the vertical axes, and the points above the red dashed lines show the significant single-nucleotide polymorphisms (SNPs) in the QTL regions. The QTLs identified are shown by black arrows. The QTLs shown in red indicate those that are overlapped for the DEC and PGWC traits.

The GWAS was conducted with the 1,224,254 SNPs to investigate possible natural variations in the chalkiness-related traits, and Manhattan plots were constructed to show the significant SNP loci. For the DEC trait, 13 QTLs were identified, which were located on chromosomes 1, 3, 4, 6, 10, 11, and 12 (Figures 2A, B); among these, four QTLs were detected on chromosome 4. The QTL *qDEC11* has the largest phenotypic variation explained (PVE) (24.89%). For the PGWC trait, a total of 9 QTLs were detected, which were located on chromosomes 3, 4, 6, 10, 11, and 12, with the PVE ranging from 11.89% to 18.18% (Figures 2C, D). The QTL *qPGWC11* has the largest PVE (18.18%).

By comparing the QTLs, five of them were identified to be common between the DEC and PGWC; these were *qDEC4.1-qPGWC4.1*, *qDEC4.4*-*qPGWC4.4*, *qDEC6.2*-*qPGWC6*, *qDEC10*-*qPGWC10*, and *qDEC11*-*qPGWC11*. Among these, both *qDEC11* and *qPGWC11* had the largest PVEs and largest numbers of significant SNP loci. Therefore, we consider *qDEC11* to be the major QTL for DEC and further analyze it.

### Identification of candidate genes for the DEC trait

**FIGURE 3:**
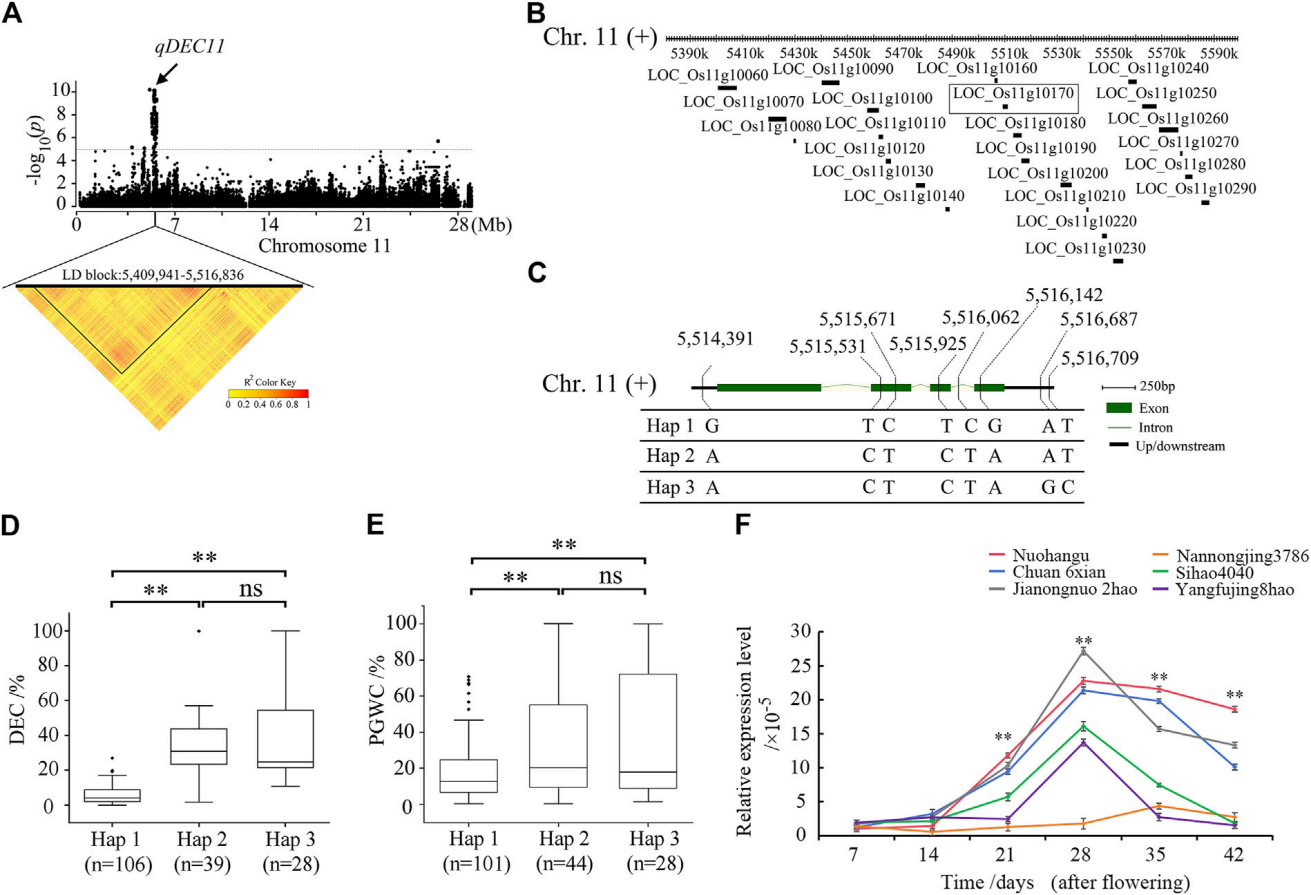
Identification of the candidate genes for the QTL *qDEC11* in rice for DEC as determined from the GWAS and gene expression analysis data. **(A)** Identification of the linkage disequilibrium (LD) block of *qDEC11*. The Y axis shows the negative-log_10_-transformed *p*-values, and the points above the red line are the significant SNPs in the QTL region. The pairwise LD was calculated with the *r* ^2^ value (determination coefficient between the SNP states). **(B)** Identification of the candidate genes in the QTL region of *qDEC11*. **(C)** Haplotypes of the *LOC_Os11g10170* gene associated with DEC in rice. **(D)** Box plots of the DEC in accessions containing different haplotypes. **(E)** Box plots of the PGWC in accessions containing different haplotypes. **(F)** Expression analysis of the candidate gene *LOC_Os11g10170* in the six materials with different AC values at different periods after flowering. The relative expressions were normalized to that of the rice UBQ gene. The error bars indicate the standard deviation, and the asterisks indicate the significant differences based on Student’s *t*-test (**p < 0.01).

To identify the candidate genes for the DEC trait, GWAS and LD analysis were conducted on chromosome 11 (Figure 3A); there were 23 candidate genes in the chromosome region of 5,388,827–5,588,827 containing *qDEC11* (Figure 3B). Based on LD analysis, the LD block region was identified as 5,409,941–5,516,836, which contained 11 candidate genes (Supplementary Table S4). From the SNP information, five of the 11 genes contained non-synonymous SNPs, among which two genes located within the locus *LOC_Os11g10170* were found by the GWAS to be significantly associated with DEC (Supplementary Tables S4, S5). The full length of the *LOC_Os11g10170* locus is 1,716 bp, including four exons and three introns (Figure 3C). The *LOC_Os11g10170* gene encodes a 387-amino acid protein. The SNPs occurred in the upstream, while the exons and introns were in the downstream sequence of the gene, resulting in the identification of three haplotypes (Figure 3C). The haplotype Hap 1 was associated with a smaller DEC, while the haplotypes Hap 2 and Hap 3 were associated with larger DEC values (Figure 3D). The SNP site at 5,515,531 changed from base T to base C at nt 649 in the cDNA sequence, resulting in a change from tryptophan (Trp) to arginine (Arg) at the amino acid 217. The SNP site at 5,516,142 changed from base G to base A at nt 1006 in the cDNA sequence, resulting in a change from aspartic acid (Asp) to asparagine (Asn) at the amino acid 336. The average DEC value of 106 accessions carrying Hap 1 was 6.19% ± 5.01%, while the corresponding average values for carrying Hap 2 and Hap 3 were 40.36% ± 37.82% and 42.14% ± 39.78%, respectively. There was no significant difference between Hap 2 and Hap 3 at *p* < 0.05. The average DEC values of Hap 2 and Hap 3 were significantly different from that of Hap 1 at *p* < 0.01 (Figure 3D). The average PGWC value of 101 accessions carrying Hap 1 was 21.82% ± 4.25%, while the corresponding average values for carrying Hap 2 and Hap 3 were 36.21% ± 12.58% and 36.60% ± 15.67%, respectively. There was no significant difference between Hap 2 and Hap 3 at *p* < 0.05. The average PGWC values of Hap 2 and Hap 3 were significantly different from that of Hap 1 at *p* < 0.01 (Figure 3E).

The qRT-PCR results showed that there were no significant expression differences between the three accessions each with high and low DEC values from days 7 to 14 after flowering (Figure 3F); however, significant differences existed between the three accessions each with high and low DEC values from days 21 to 42 after flowering (Figure 3F). The expressions of *LOC_Os11g10170* in the accessions with high DEC values showed gradual increases from days 21 to 28, reaching a maximum at 28 days. After 28 days, the expressions of *LOC_Os11g10170* started declining gradually (Figure 3F). These results indicate that decreasing the expression of *LOC_Os11g10170* could reduce the DEC.

Given that gene *LOC_Os11g10140* contains the highest *p*-value SNP and *LOC_Os11g10090* contains the premature stop codon SNP, their haplotype analyses were carried out. For gene *LOC_Os11g10140*, the transcript length was 1,017 bp, including four coding exons and three introns (Supplementary Figure S1A) as well as three haplotypes (Supplementary Figure S1A). The average DEC value of 83 accessions carrying Hap 1 was 14.70% ± 5.67%, while the corresponding average values for carrying Hap 2 and Hap 3 were 15.69% and 16.25%, respectively. There were no significant differences among the three haplotypes (Supplementary Figure S1B). The average PGWC value of 70 accessions carrying Hap 1 was 28.52% ± 9.83%, while the corresponding average values for carrying Hap 2 and Hap 3 were 26.59% and 28.90%, respectively. There were no significant differences among the three haplotypes (Supplementary Figure S1C).

For the gene *LOC_Os11g10090*, the transcript length was 2,046 bp, including four coding exons and six introns (Supplementary Figure S2A) as well as three identified haplotypes (Supplementary Figure S2A). The average DEC value of 76 accessions carrying Hap 1 was 14.97% ± 9.74%, while the corresponding average values for carrying Hap 2 and Hap 3 were 13.22% and 14.64%, respectively. There were no significant differences among the three haplotypes (Supplementary Figure S2B). The average PGWC value of 73 accessions carrying Hap 1 was 28.89% ± 7.48%, while the corresponding average values for carrying Hap 2 and Hap 3 were 26.44% and 28.43%, respectively. There were no significant differences among the three haplotypes (Supplementary Figure S2C).

## Discussion

Rice quality is a complex trait, and endosperm opacity is one of the main attributes that determines the appearance quality of rice grains. Previous studies have shown that chalkiness is the result of interactions between the genetic background and environmental factors (Liao and Zhang, 2015; Zhou et al., 2016). The main environmental factors include the ecological environment, cultivation practices, and climate conditions (Xie et al., 2017). To reduce the influence of the environmental factors on phenotype, the chalkiness-related traits of 173 accessions were investigated in two environments, which showed great genetic variations. The CV ranged from 96.51% to 214.18%. These results provide a material basis for breeding to improve the chalkiness-related traits.

In this study, a total of 22 QTLs were detected, of which 13 controlled the DEC and nine controlled the PGWC traits. Among these QTLs, four common QTLs were identified for both DEC and PGWC, which were located on chromosomes 4, 6, 10, and 11 (Figure 2). By combining the information reported at the Gramene (http://www.gramene.org/markers/), BLAST (http://blast.ncbi.nlm.nih.gov/Blast.cgi), and China Rice Data Center database (http://www.ricedata.cn/gene/list/1499.htm) websites, the QTLs identified in this study were compared with those reported previously for controlling the DEC/PGWC. We compared the results identified in this study with those reported previously by Misra et al. (2019, 2021) and Sachdeva et al. (2024) and found that none of the identified loci were common, which could be related to the large differences in the geographical origins and characteristics of the varieties used. The varieties used by Misra et al. (2019, 2021) and Sachdeva et al. (2024) included indica, aus, tropical japonica from the IRRI South Asia Regional Center, Varanasi, Uttar Pradesh, India, whereas the materials used in this study included indica mainly from South China, East China, Central China, Vietnam, Philippines, and Indonesia as well as temperate japonica rice from East China, North China, and Northeast China. The results obtained in this study were also compared with those reported previously by Xin et al. (2022), and none of the loci identified were common; this may be attributed to the fact that the genetic backgrounds of the materials used were very different. The position ranges of six of the QTLs overlapped with the flanking regions of 12 of the QTLs reported previously, and the remaining 16 QTLs were newly identified in this study (Supplementary Table S6). Among the 16 QTLs newly identified herein, the QTL *qDEC11* that is colocated with *qPGWC11* had the largest PVE (24.89%) and highest number of significant SNP loci (225).

One GWAS signal that is significantly associated with DEC was detected to nearly single-gene resolution. In the LD block region 5,409,941–5,516,836 of chromosome 11, *LOC_Os11g10170* is a newly identified candidate gene (Figure 3). The full length of *LOC_Os11g10170* is 1,716 bp, including four exons and three introns; this gene encodes a 387 amino acid protein. The base T-to-C and G-to-A non-synonymous mutations in the cDNA sequence of *LOC_Os11g10170* result in the high DEC phenotype (Figure 3). The qRT-PCR results showed that significant expression differences existed between the three accessions each with the highest and lowest DEC values from days 21 to 42 after flowering, reaching a maximum at 28 days (Figure 3F). However, we also suspected that the differences in these gene expression levels could be due to changes in the amino acids, leading to functional differences. Tryptophan produces indole-3-pyruvate in the presence of tryptophan transaminase, and indole-3-pyruvate produces auxin in the presence of YUCs. The change from tryptophan to arginine caused by the change in the base prevents the latter reaction, which affects the difference in gene expression level. The results of haplotype and qRT-PCR analyses indicate that *LOC_Os11g10170* is the candidate gene for the DEC.

Auxin is an important plant hormone that plays a vital role in plant growth and development, while also participating in the formation and development of plant organs (Cheng et al., 2006, 2007). Indole-3-acetic acid (IAA) is the major auxin in plants, and the indole-3-pyruvate acid (IPA) pathway is the major tryptophan-dependent pathway for auxin biosynthesis in plants. For this pathway, the conversion of IPA to IAA by flavin monooxygenase encoded by the *Yucca* gene is the rate-limiting step (Hofmann et al., 2011; Stepanova et al., 2011). According to the website of the Rice Genome Annotation Project, the product encoded by *LOC_Os11g10170* is flavin monooxygenase. Mashiguchi et al. (2011) demonstrated that *Yucca*-encoded flavin monooxygenase can catalyze the direct conversion of IPA to IAA based on gene function analysis, *in vitro* experiments, and isotope tracing observations. In the *Arabidopsis thaliana* genome, there are 11 *Yucca* genes that are mainly expressed in the apical meristems, young primordia, vascular tissues, and seeds (Cheng et al., 2006). In maize, the mutation of the *Yucca1* gene was shown to result in sparse inflorescence and fewer branches (Gallavotti et al., 2008). In the rice genome, there are 14 *Yucca* homologous genes (Stepanova et al., 2011). *OsYUCCA1* is located on chromosome 1 and affects auxin biosynthesis (Yamamoto et al., 2007). The *OsFMO(t)* gene located on chromosome 3 regulates IAA biosynthesis possibly locally and plays an important role in the formation of local IAA concentrations that are critical for regulating normal growth and development in rice (Yi et al., 2013). The *OsFMO1* gene located on chromosome 9 can affect auxin synthesis, signal transduction, polar transport, and growth and development in plants (Liu et al., 2019). Grain filling is an important physiological process in rice growth; therefore, we hypothesize that *LOC_Os11g10170* encoding flavin monooxygenase may inhibit or accelerate the transport of photosynthetic products through the phloem to the endosperm cells in the form of sucrose during grain filling to affect starch synthesis, resulting in different degrees of chalkiness among the accessions. This provides a possible scientific basis for further research on the *LOC_Os11g10170* gene with regard to the developmental growth and quality of rice.

## Data Availability

The data presented in the study are deposited in the Sequence Read Archive (SRA), NCBI accession number PRJNA554986. The names of the repositories and accession numbers can be found in the article/Supplementary Material.
