# Supplementary material for: Genome-wide association mapping of quantitative trait loci for chalkiness-related traits in rice (Oryza sativa L.)
Source: Front Genet. 2024 Jul 10;15:1423648. doi: 10.3389/fgene.2024.1423648 (PMC11266141; doi:10.3389/fgene.2024.1423648)
Supplement: Supplementary file 1 [file Table4.DOC]

**Table S4.** Candidate gene annotation in the region 5.40–5.52 Mb associated with degree of endosperm chalkiness.

| Number | MSU ID | Position | Annotation |
| --- | --- | --- | --- |
| 1 | LOC_Os11g10060 | 5,400,115-5,407,497 | Transcriptional corepressor SEUSS |
| 2 | LOC_Os11g10070 | 5,420,328-5,427,177 | Transcriptional corepressor SEUSS |
| 3 | LOC_Os11g10080 | 5,436,885-5,437,421 | Expressed protein |
| 4 | LOC_Os11g10090 | 5,441,804-5,448,578 | Transposon protein |
| 5 | LOC_Os11g10100 | 5,460,201-5,464,655 | MAPK kinase |
| 6 | LOC_Os11g10110 | 5,464,851-5,466,306 | Expressed protein |
| 7 | LOC_Os11g10120 | 5,467,512-5,469,283 | Expressed protein |
| 8 | LOC_Os11g10130 | 5,479,654-5,482,999 | MYB family transcription factor |
| 9 | LOC_Os11g10140 | 5,491,381-5,493,004 | Flavin monooxygenase |
| 10 | LOC_Os11g10160 | 5,511,173-5,512,067 | Expressed protein |
| 11 | LOC_Os11g10170 | 5,514,584-5,516,300 | Flavin monooxygenase |
