# Supplementary material for: Genome-wide association mapping of quantitative trait loci for chalkiness-related traits in rice (Oryza sativa L.)
Source: Front Genet. 2024 Jul 10;15:1423648. doi: 10.3389/fgene.2024.1423648 (PMC11266141; doi:10.3389/fgene.2024.1423648)
Supplement: Supplementary file 2 [file Table6.DOC]

**Table S6.** The results of QTLs detected in this study overlapped with the QTLs/Genes reported previously.

| QTL name | Chr. | Position range /bp | Known QTL^a^/  association locus^a^ | Flanking region /bp^a^ | Reference |
| --- | --- | --- | --- | --- | --- |
| *qDEC1.1* | 1 | 30,243,484-30,443,484 | *qDEC-1b* | 29,446,995-34,471,466 | Wan et al. 2005 |
|  |  |  | *qPGWC-1* | 29,446,995-34,471,466 | Wan et al. 2005 |
| *qDEC1.2* | 1 | 32,387,680-32,587,680 | *qDEC-1b* | 29,446,995-34,471,466 | Wan et al. 2005 |
|  |  |  | *qPGWC-1* | 29,446,995-34,471,466 | Wan et al. 2005 |
| *qDEC3.2* | 3 | 14,902,348-15,102,348 | *PGC3.6* | 15,024,656-15,024,656 | Misra et al. 2021 |
| *qDEC4.4* | 4 | 28,945,765-29,145,765 | *PGC4.4* | 29,023,839-31,212,801 | Misra et al. 2021 |
| *qDEC6.1* | 6 | 2,532,094-2,732,094 | *-* | 2,260,721-3,536,009 | Tan et al. 2000 |
|  |  |  | *qPGWC-6a* | 2,686,204-3,456,103 | Li et al. 2003 |
|  |  |  | *qPGWC-6b* | 2,260,721-6,485,558 | Zhou et al. 2009 |
|  |  |  | *qPGWC6* | 642,735-2,261,604 | Zheng et al. 2021 |
| *qPGWC4.4* | 4 | 29,110,165-29,310,165 | *PGC4.4* | 29,023,839-31,212,801 | Misra et al. 2021 |
|  |  |  | *-* | 2,260,721-3,536,009 | Tan et al. 2000 |

^a^The gene name, QTL name and the physical position (bp) was inferred from the database of Gramene website (http://www.gramene.org/markers/), BLAST (http://blast.ncbi.nlm.nih.gov/Blast.cgi) and the China Rice Data Center database (http://www.ricedata.cn/gene/list/1499.htm).
