# Supplementary material for: Genome-wide association mapping of quantitative trait loci for chalkiness-related traits in rice (Oryza sativa L.)
Source: Front Genet. 2024 Jul 10;15:1423648. doi: 10.3389/fgene.2024.1423648 (PMC11266141; doi:10.3389/fgene.2024.1423648)
Supplement: Supplementary file 4 [file Table3.DOC]

**Table S3.** The sequences of primers used for qRT-PCR.

| Gene name | Forward primer sequence (5’-3’) | Reverse primer sequence (5’-3’) |
| --- | --- | --- |
| UBQ | ACCCTGGCTGACTACAACATC | AGTTGACAGCCCTAGGGTG |
| LOC_Os11g10170 | AGGGCTAAATGTGACAGCAG | GTGCCAACATCAATAACGGC |
