# Supplementary figures and images for: Genome-wide association mapping of quantitative trait loci for chalkiness-related traits in rice (Oryza sativa L.)
Source: Front Genet. 2024 Jul 10;15:1423648. doi: 10.3389/fgene.2024.1423648 (PMC11266141; doi:10.3389/fgene.2024.1423648)

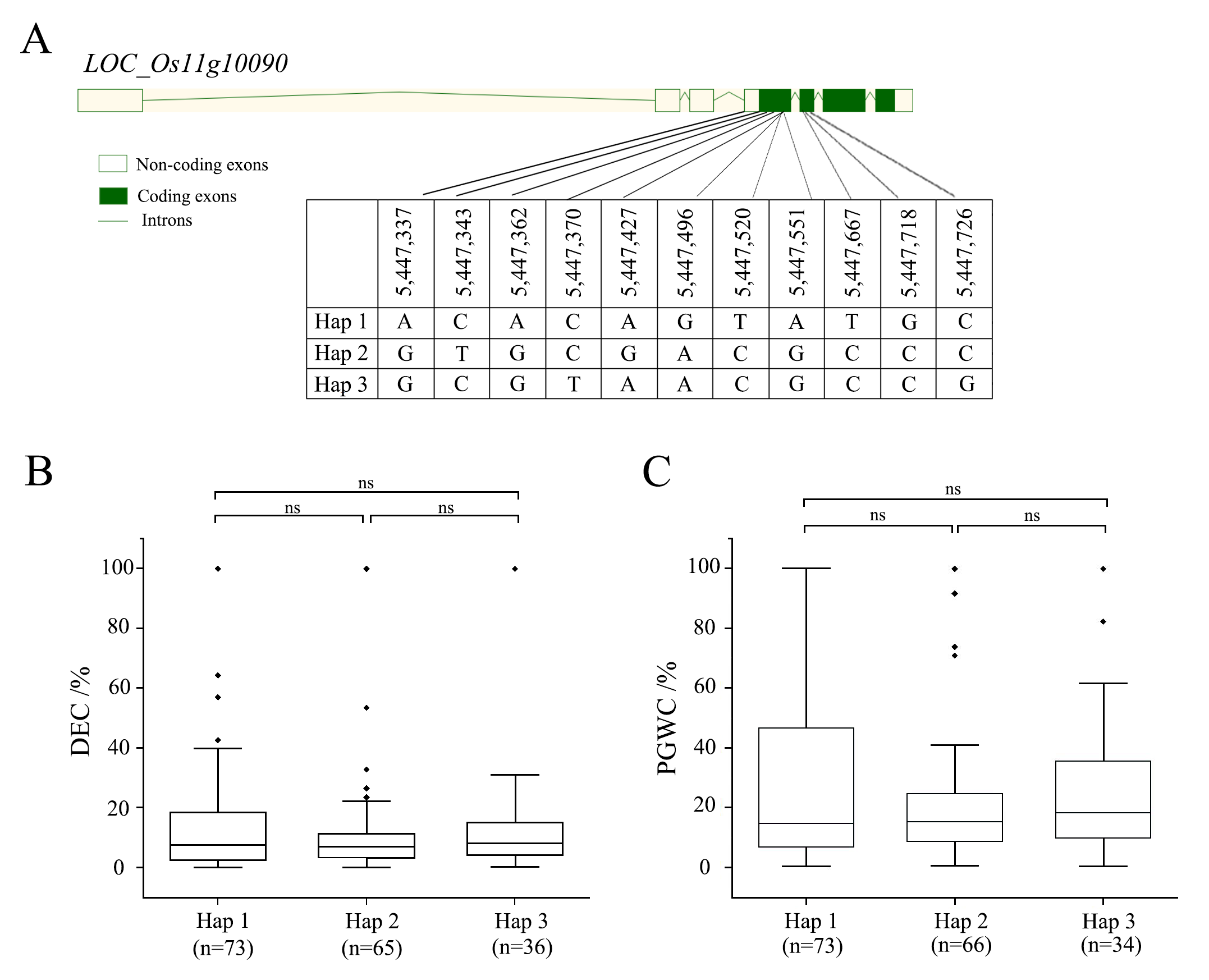

Supplement: Supplementary file 6 [file Image2.TIF]

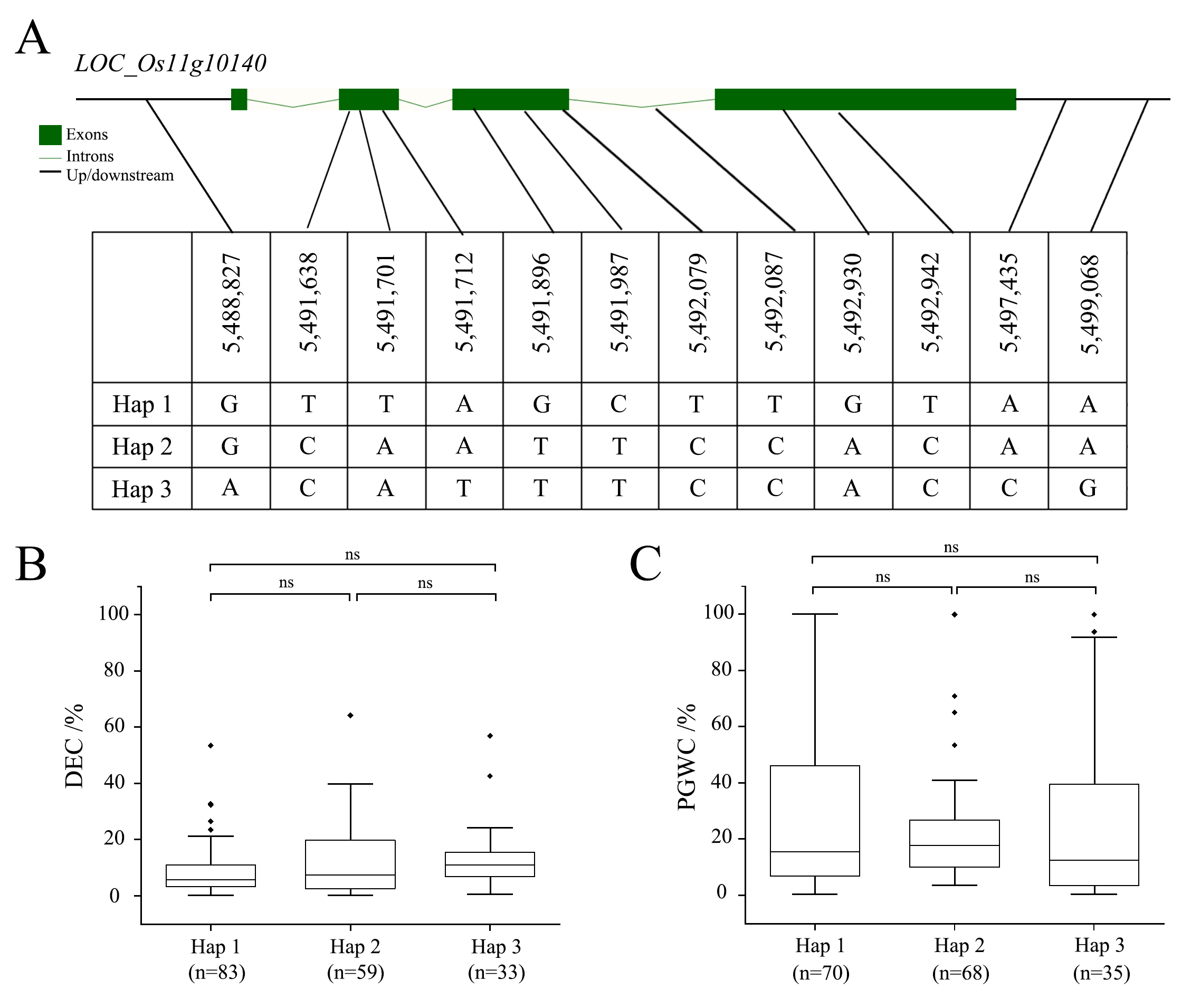

Supplement: Supplementary file 7 [file Image1.TIF]
