# Supplementary material for: Genome-wide association mapping of quantitative trait loci for chalkiness-related traits in rice (Oryza sativa L.)
Source: Front Genet. 2024 Jul 10;15:1423648. doi: 10.3389/fgene.2024.1423648 (PMC11266141; doi:10.3389/fgene.2024.1423648)
Supplement: Supplementary file 8 [file Table1.DOC]

**Table S1**. A list of QTLs controlling DEC and PGWC published so far.

| Traits | Chromosomes | | | | | | | | | | | | References |
| --- | --- | --- | --- | --- | --- | --- | --- | --- | --- | --- | --- | --- | --- |
|  | 1 | 2 | 3 | 4 | 5 | 6 | 7 | 8 | 9 | 10 | 11 | 12 |  |
| DEC /% | 1 |  |  |  | 2 | 2 | 1 |  |  | 1 |  |  | Tan et al. 2000 |
|  |  |  |  |  |  |  |  | 2 |  |  | 2 | 1 | Zeng et al. 2002 |
|  |  |  | 1 |  | 1 | 2 |  |  |  |  |  |  | Li et al. 2003 |
|  |  |  |  |  |  |  |  |  |  |  |  | 1 | Li et al. 2004 |
|  | 2 | 1 |  |  |  |  |  | 1 | 1 |  |  |  | Wan et al. 2005 |
|  |  |  |  |  |  |  |  |  | 1 |  |  | 1 | Liu et al. 2007 |
|  | 2 | 2 |  | 4 |  | 3 | 1 | 2 | 2 |  | 2 | 1 | Tao et al. 2015 |
|  |  |  |  |  | 2 | 1 |  |  |  |  |  |  | Chen et al. 2022 |
| Total | 5 | 3 | 1 | 4 | 5 | 8 | 2 | 5 | 4 | 1 | 4 | 4 |  |
|  |  |  |  |  |  |  |  |  |  |  |  |  |  |
| PGWC /% | 1 |  | 1 |  | 1 | 2 |  | 1 |  |  |  | 1 | Li et al. 2003 |
|  | 1 |  |  |  |  |  |  | 1 | 1 |  |  |  | Wan et al. 2005 |
|  |  |  |  |  | 1 |  |  | 1 | 1 |  | 1 | 1 | Liu et al. 2007 |
|  | 2 |  | 1 | 1 |  | 3 |  |  |  |  | 1 |  | Zhou et al. 2009 |
|  |  |  |  |  | 2 |  |  |  |  |  |  |  | Wang et al. 2011 |
|  |  | 1 | 1 |  |  | 1 |  |  |  |  |  |  | Zheng et al. 2012 |
|  |  | 2 |  | 1 | 2 | 2 | 1 |  | 2 |  |  | 1 | Peng et al. 2014 |
|  |  | 2 | 1 |  |  | 1 |  | 2 |  |  |  | 1 | Ma et al. 2015 |
|  | 2 | 3 | 1 | 4 | 1 | 1 |  |  | 3 |  | 1 | 1 | Tao et al. 2015 |
|  | 2 | 4 | 2 | 1 | 3 | 6 |  |  | 1 | 2 |  |  | Misra et al. 2021 |
| Total | 8 | 12 | 7 | 7 | 10 | 16 | 1 | 5 | 8 | 2 | 3 | 5 |  |

DEC: the degree endosperm chalkiness; PGWC: the percentage of grains with chalkiness.
